# Evaluation of Relationships between Growth Rate, Tree Size, Lignocellulose Composition, and Enzymatic Saccharification in Interspecific *Corymbia* Hybrids and Parental Taxa

**DOI:** 10.3389/fpls.2016.01705

**Published:** 2016-11-18

**Authors:** Adam L. Healey, David J. Lee, Jason S. Lupoi, Gabriella Papa, Joel M. Guenther, Luca Corno, Fabrizio Adani, Seema Singh, Blake A. Simmons, Robert J. Henry

**Affiliations:** ^1^Queensland Alliance for Agriculture and Food Innovation, University of QueenslandSt. Lucia, QLD, Australia; ^2^Forest Industries Research Centre, University of the Sunshine CoastMaroochydore, QLD, Australia; ^3^Forestry & Biosciences, Agri-Science Queensland, Department of Agriculture and FisheriesGympie, QLD, Australia; ^4^Prozess TechnologieSt. Louis, MO, USA; ^5^Joint BioEnergy Institute, Lawrence Berkeley National LaboratoryEmeryville, CA, USA; ^6^Biological and Engineering Sciences Center, Sandia National LaboratoriesLivermore, CA, USA; ^7^Gruppo Ricicla – Biomass and Bioenergy Laboratory, DiSAA, University of MilanMilan, Italy

**Keywords:** *Corymbia*, biofuels, eucalypt, saccharification, growth rate, lignin, glucan, xylan

## Abstract

In order for a lignocellulosic bioenergy feedstock to be considered sustainable, it must possess a high rate of growth to supply biomass for conversion. Despite the desirability of a fast growth rate for industrial application, it is unclear what effect growth rate has on biomass composition or saccharification. We characterized Klason lignin, glucan, and xylan content with response to growth in *Corymbia* interspecific F1 hybrid families (HF) and parental species *Corymbia torelliana* and *C. citriodora* subspecies *variegata* and measured the effects on enzymatic hydrolysis from hydrothermally pretreated biomass. Analysis of biomass composition within *Corymbia* populations found similar amounts of Klason lignin content (19.7–21.3%) among parental and hybrid populations, whereas glucan content was clearly distinguished within *C. citriodora* subspecies *variegata* (52%) and HF148 (60%) as compared to other populations (28–38%). Multiple linear regression indicates that biomass composition is significantly impacted by tree size measured at the same age, with Klason lignin content increasing with diameter breast height (DBH) (+0.12% per cm DBH increase), and glucan and xylan typically decreasing per DBH cm increase (-0.7 and -0.3%, respectively). Polysaccharide content within *C. citriodora* subspecies *variegata* and HF-148 were not significantly affected by tree size. High-throughput enzymatic saccharification of hydrothermally pretreated biomass found significant differences among *Corymbia* populations for total glucose production from biomass, with parental *Corymbia torelliana* and hybrids HF-148 and HF-51 generating the highest amounts of glucose (~180 mg/g biomass, respectively), with HF-51 undergoing the most efficient glucan-to-glucose conversion (74%). Based on growth rate, biomass composition, and further optimization of enzymatic saccharification yield, high production *Corymbia* hybrid trees are potentially suitable for fast-rotation bioenergy or biomaterial production.

## Introduction

Due to the un-sustainable nature and detrimental effects of fossil fuels on climate change, there is increased interest for the development of renewable plant-based alternatives for energy production (Simmons et al., 2008; Furtado et al., 2014). Advanced future biofuels will likely derive from non-edible feedstocks, where structural polysaccharides provide the main substrate for biochemical conversion into fuel. Lignocellulose, or woody biomass, is a potential feedstock for bioenergy production considering its availability, cost of production and scale at which it can be generated (Wang et al., 2012); however, lignocellulose’s natural recalcitrance to deconstruction prevents the economic conversion of biomass into fuel (Studer et al., 2011).

Enzymatic hydrolysis of structural polysaccharides is the most critical and costly aspect of biofuel production due to the structure and chemistry of the plant cell wall (Yu et al., 2011). The major structural components of lignocellulose (cellulose, hemicellulose, lignin) each contribute to biomass recalcitrance (Wang et al., 2016), but in woody biomass lignin has been demonstrated to contribute most negatively to saccharification, as its structure prevents enzymatic access to cellulose and non-specifically binds and immobilizes cellulase enzymes (Leu and Zhu, 2013). Additionally, lignin covalently bonds with hemicellulose, creating lignin-carbohydrate complexes that further inhibit saccharification (Yang and Wyman, 2004). Hemicellulose also affects efficient saccharification of lignocellulose due to its composition of difficult to ferment 5′-carbon sugars and its effect on the porosity of biomass through its cross-linkages with cellulose (Lange, 2007; Nigam and Singh, 2011). Despite being the main target for conversion to biofuel, cellulose also resists efficient hydrolysis due to its crystalline structure resulting in hydrophobic macrofibrils with limited reactive surface area and extensive hydrogen bonding (Yang et al., 2011; Mizrachi et al., 2012; Zhang Y. et al., 2015).

In order for a lignocellulose feedstock to be considered as a sustainable option for biofuel production, it must possess a growth rate suitable for economic harvesting. Eucalypt trees are an ideal candidate as a biofuel crop based on their established silviculture practices, global deployment, rapid growth in marginal soils and wide range of rainfall conditions, and genomic resources dedicated to wood formation and environmental resistances (Shepherd et al., 2011; Grattapaglia et al., 2012; Myburg et al., 2014; Healey et al., 2015). In Queensland, Australia, due to climate and environmental stresses, *Corymbia citriodora* subspecies *variegata* (CCV) is the most widely harvested hardwood tree, based on its form, wood quality and tolerances to variable soils, drought, pests, and disease (Lee et al., 2010). Examination of pulpwood traits across multiple trial sites indicate *C. citriodora* subspecies *variegata* is also well-suited for pulp and paper production based on predicted Kraft pulp yield (55% pulp per wood volume) and density (756 kg/m^3^), and moderate trait heritability (0.3 and 0.5, respectively) across multiple trial sites (Brawner et al., 2012). Tree improvement programs have also demonstrated the potential of F1 interspecies *Corymbia* hybrids (*C. torelliana* × *C. citriodora* subspecies *variegata*), combining desirable forestry traits (form, wood quality, vegetative propagation) within a single genetic background and possessing a superior growth rate [127–287%, diameter breast height (DBH)] as compared to either parental taxa (Lee et al., 2009).

While a high rate of growth is desirable for a variety of forestry applications, it is unclear what effect growth rate plays in altering biomass composition or affecting enzymatic saccharification. Transgenic manipulation of woody biomass has demonstrated that alteration of growth rate influences biomass composition and vice versa. Overexpression of a growth hormone precursor to gibberellin in transgenic poplar trees significantly improved growth rate in seedlings and biomass production within stem tissue. Additionally, transgenic lines also had longer and more numerous xylem fibers, which are commercially desirable for producing wood pulp with higher tensile strength (Eriksson et al., 2000). Disruption of lignin biosynthesis in transgenic poplar hybrids can negatively impact growth form and habit, where transgenic lines with significantly reduced lignin content resulted in brown discolored xylem tissue and dwarfed trees with reduced height, DBH, and growth rate (Leplé et al., 2007). Without lignin reinforcement during stem growth and thickening, xylem fibers are prone to collapse and cavitation being unable to withstand water pressures required for long distance transport (Kawaoka et al., 2006; Coleman et al., 2008; Voelker et al., 2011). Similarly, transgenic manipulation of cellulose biosynthesis can negatively impact growth rate and alter biomass composition. Joshi et al. (2011) demonstrated that the up-regulation of cellulose synthase (*Ces*A8) inadvertently caused sense silencing of the native *Ces*A and transgene, producing trees with little cellulose (10% dry weight) and a proportionate increase in lignin content (35%) and non-cellulosic polysaccharides (55%). As there is a strong interaction between growth and biomass composition, increased biomass production could negatively impact biofuel conversion processes if carbon resources shift toward xylem lignification. Small percentage increases in lignin content greatly affect substrate access for cellulases (Gressel, 2008), resulting in biomass that is inefficiently deconstructed and hydrolyzed. The aim of this study was therefore to examine the effect of growth rate on the main components of lignocellulose composition (glucan, xylan, Klason lignin) and their subsequent effect on enzymatic saccharification within commercial *Corymbia* interspecies hybrids and parental taxa. Given the significant impact each biomass component contributes toward saccharification, changes in biomass composition that occur in response to a high rate of growth will inform optimal harvest size for industrial use of woody biomass.

## Materials and Methods

### Sample Collection

Populations of *Corymbia torelliana*, *C. citriodora* subspecies *variegata* and interspecies controlled-cross F1 hybrids (HF) from the Queensland Department of Agriculture and Fisheries (DAF) from the Amamoor trial site located near Gympie, Queensland were measured for DBH at age 13 years. A minimum of five trees per population per size class (if available) were selected at random for biomass extraction (**Table 1**). Wood frass was collected at a height of 1.3 m on the north-facing side of the tree, adjusting for knots and tension wood, with a 16 mm wood boring bit and a modified funnel. Approximately 2–4 g of sapwood frass was collected per tree, and was air-dried in a paper bag in an air-conditioned room for 14 days and shipped to the Joint BioEnergy Institute in Emeryville, CA, USA. Given the large variation in wood particle size, size reduction was performed prior to compositional analysis and saccharification by placing samples into 2 mL polyethylene vials (Sarstedt VWR 72.609.001) with three ceramic beads (yttrium stabilized zirconia, 5 mm^1^), and grinding for 5 min (2.5 min grind, 60s rest, repeat) using the Joint BioEnergy Institute Biomass Preparation System Robot, created by Labman Automation Ltd. (North Yorkshire, UK). The ground biomass was sieved using a 40 mesh (0.4 mm) filter to further reduce particle size variation. Samples were placed into re-closable antistatic bags (RoyalBag-#1646) prior to dispensing.

**Table 1:** Size categories of *Corymbia* hybrid and parental trees randomly sampled at age 13 years, from the Amamoor plantation site, located near Gympie, Australia.

Size Class	DBH (cm)	Number of Trees
Small (S)	6.4–12.4	36
Medium (M)	12.5–20.3	39
Large (L)	18.0–24.1	38
Extra Large (XL)	27.0–32.2	23 (excluding *Corymbia citriodora* subspecies *variegata* and HF-151)
Extra Extra Large (XXL)	36.0–40.6	5 (HF-148 only)

### Compositional Analysis

Compositional Analysis was carried out using National Renewable Energy Laboratory (NREL) methods (Sluiter et al., 2011) with minor modifications. Using an analytical balance, 100 mg (±5 mg) of biomass was dispensed in triplicate into 100 mL serum bottles. One mL of 72% sulfuric acid was added to each biomass aliquot, along with a small plastic coated stir-bar to aid in biomass disruption. Samples were macerated with a glass rod, covered with aluminum foil, and placed in a 30°C climate controlled room for 1 h incubation with mixing every 15 min. After incubation, sulfuric acid was diluted to 4% with ultrapure water to a final volume of 29 mL. Glass bottles were closed with a rubber stopper and clamped shut using aluminum crimp top seals (Sigma-Aldrich). Samples were then autoclaved for 1 h (121°C) and allowed to cool before opening. Prior to vacuum filtration for Klason lignin quantification, 1 mL of decanted lysate was collected for structural sugars quantification by High Performance Liquid Chromatography (HPLC). The remaining supernatant was vacuum filtered through a 25 mL crucible (Coors #60531), previously heated at 105°C for a minimum of 1 h and cooled to room temperature for 30 min in a desiccator before weighing. The serum bottles were washed with deionized water to remove any particles clinging to the glass wall inner surface and the solution vacuum filtered free of acid. Crucibles were dried for a minimum duration of 6 h at 105°C, and then cooled to room temperature for 30 min in a desiccator before crucible weights were collected.

The crucibles containing the dried residues were placed in a furnace and pyrolyzed using a modified pyrolysis protocol consisting of a 2 h minimum incubation at 575°C and hold at 105°C. The modified protocol was introduced after no observable or measurable ash content was present after analysing a representative group of *Corymbia* biomass samples. Lignin weight was determined as percent dry biomass as follows: % Klason lignin = (Final weight after incubation at 105°C/Initial weight of samples)^∗^100.

### Structural Sugars Quantification

One hundred and fifty microliters of extracted lysate was filtered through a 96 well 0.45 μm filter plate (Whatman, 7700-1301^2^) by centrifugation (3000 × *g* for 3 min) into a 96 well 200 μL PCR plate (Bio-Rad, Hercules, CA, USA, HSP9601). PCR plates were sealed using a pierceable aluminum heat seal (Agilent 06644-001), applied using a PlateLoc sealer (175°C, 4 s; Agilent Technologies). HPLC was performed using an Agilent 1260 Infinity system (Agilent, Santa Clara, CA, USA) with a Bio-Rad 87H 300 mm × 7.8 mm Aminex column (Bio-Rad, Hercules, CA, USA) with a cation H guard column. The refractive index detector was held at 35°C. The eluent was 4 mM isocratic sulfuric acid, prepared with HPLC grade water (Honeywell, Morristown, NJ, USA) and 98% sulfuric acid (Millipore, Billerica, MA, USA). Each analytical run used an eluent flow rate of 0.6 mL/min, and temperature set to 60°C for 16 min. Sugar calibration standards were prepared and diluted to create an eight-point calibration curve, 0.015–2.0 mg/mL for cellobiose, xylose, and arabinose, and 0.03–4.0 mg/mL for glucose. Standards were run at the beginning, middle and end of each 96 well plate. De-ionized water blanks were inserted into the sample queue before and after each run of standards. The concentrations of glucose, xylose, cellobiose, and arabinose in the samples were calculated using the Chemstation software package and by integrating the area under each sugar peak. Glucan content was calculated as: Glucan content (%) = ((glucose concentration [mg/mL]^∗^*V*^∗^0.9)/*m*)^∗^100 where *V* is the volume of hydrolysis liquid (mL), *m* is the mass of the sample (mg) and 0.9 is the conversion factor for glucose to glucan.

### Enzymatic Saccharification

Ground biomass samples were dispensed for saccharification using the Joint BioEnergy Institute Biomass Preparation System Robot at a target mass of 10 (±0.5) mg of biomass per well into a 2 mL 96 deep-well polypropylene block (Corning Costar 3961). Samples were dispensed in duplicate between two separate blocks. Biomass extraction was conducted by adding 1 mL of 80% ethanol into each well using a Biomek FX liquid-handling robot with an AP96 multichannel pod (Beckman, Coulter, Brea, CA, USA). Each 96 well block was sealed with a peelable heat seal and incubated at 37°C within a thermostatically controlled room for 24 h with shaking at 150 RPM. Ethanol and extractives were removed from each well using ultrapure water washes until the ethanol concentration was less than 1% in a final volume of 820 μL per well. For hydrothermal pretreatment, plates were sealed shut with a rubber mat and metal clamp and autoclaved for 1 h at 121°C. Sample de-starching prior to hydrothermal pretreatment and saccharification was found unnecessary as previous experiments, found no additional sugar release from *Corymbia* biomass after amylase treatment (data not shown).

Saccharification was performed using the Biomek FX to dispense 180 μL of enzyme solution (8.2:1 v:v ratio of Cellic CTec2:HTec2; Novozymes, Franklinton, NC, USA) and citrate buffer (pH 5.0) to a final concentration of 100 mM. Enzyme loading per well was approximately 60 mg/g glucan, chosen empirically to maximize the observed differences between eucalypt samples with known low/high glucose saccharification yields (data not shown). Each block was sealed with a peelable seal and incubated at 55°C for 48 h without agitation in a Thermos oven (Thermo Scientific, Waltham, MA, USA). After 48 h, the blocks were centrifuged for 3 min at 3000 × *g*, and then placed onto the Biomek FX robot which transferred 100 μL of solution into a Whatman 0.45 μm filter plate. The samples were centrifuged (3 min at 3000 × *g*) into a 96 well Bio-Rad PCR plate and sealed with a pierceable aluminum heat seal and placed at -80°C. Prior to running on the HPLC, plates were thawed overnight at 4°C and diluted 5X in 100 mM citrate buffer in a new 96 well PCR plate, sealed with a pierceable aluminum seal. Glucose quantification using the HPLC was conducted as previously described for compositional analysis.

The conversion of cellulose to glucose in the enzymatic hydrolysis was determined by the ratio of the glucose concentration that was released during enzymatic hydrolysis to the total glucose in the substrate and was calculated using formula:

$$Theoritical\,\;conversion\,\;of\,\;glucan\,\;to\,\;glu\,\cos e\,\;\left(\%\right)={\left(\frac{glu\,\cos e*V*0.9}{glucancontent(\%)*m}\right)}^{*}100$$

where glucose is glucose concentration in the enzymatic hydrolysis liquor (mg/mL); *V* is volume of enzymatic hydrolysis liquor (mL); *m* is mass of sample (mg).

### Data Analysis

Biomass composition was calculated from the mean of three technical replicates if the replicate’s coefficient of variation (CV) was <20%. In instances where CV was greater than 20%, the mean of two replicates was used. If the CV from all technical replicates was >20%, all data points were excluded and treated as missing data. Saccharification values (total glucose production and conversion efficiency) were calculated from the mean of two technical replicates if sample CV was <20%. In instances where CV was >20%, both data points were excluded and treated as missing data. Multiple linear regression (MLR) was conducted for each biomass compositional trait and saccharification yield using R Studio (version 3.0.2), with preliminary analysis to ensure that there was no violation of the assumption of normality, linearity, and multicollinearity. Given that XL and XXL trees (**Table 1**) could not be found in each *Corymbia* population, these additional size classes were initially excluded from the regression models.

#### Biofuel Trait Model

$$\gamma_{i}=\mu +S_{j}+D+S_{j}\times D+\varepsilon_{j}$$

where γ represents dependent biomass component (i = Klason lignin, glucan and xylan (% biomass)), μ represents the intercept of the model, *S* represents the various *Corymbia* populations (j), *D* represents measured DBH in cm *S*_j_ × *D* is the interaction term between species and size and 𝜀 is the vector for random residual error.

#### Total Glucose Production and Glucan Conversion

$$\gamma_{i}=\mu +S_{j}+L+\varepsilon_{j}$$

where γ represents enzymatic saccharification [i = total glucose production (mg glucose/g biomass), theoretical glucan conversion to glucose (%)], μ represents the intercept of the model, *S* represents the various *Corymbia* populations (j), *L* is Klason lignin content (% biomass) of the biomass, and 𝜀 is the vector for random residual error.

## Results

### Biomass Composition

Klason lignin, glucan, and xylan content, as well as glucose yield (mg/g raw biomass) and (%) conversion efficiency are summarized in **Table 2**. The population standard deviations (SDs) for each trait are described below in the text, whereas experimental standard error (SE) is provided in **Table 2**.

**Table 2:** Biomass composition (% dry weight basis), glucose yield (mg/g raw biomass) and (%) conversion efficiency obtained from enzymatic hydrolysis of 13-year-old *Corymbia* hybrids and parental taxa among small, medium and large size trees.

Population	Genetic Background	Lignin	Glucan	Xylan	Glucose Production	Glucose Conversion (%)
*Corymbia torelliana* (CT)	Mixed Provenances	20.4 (0.4)	35 (2)	13.4 (0.7)	189 (8)	62 (5)
*Corymbia citriodora* subspecies *variegata* (CCV)	W	20.9 (0.6)	52 (2)	20.2 (0.4)	150 (11)	33 (3)
HF-148	CT2-011 × CV2-046	21.0 (0.4)	60.0 (0.9)	20.1 (0.3)	188 (8)	35 (2)
HF-153	CT2-011 × CV2-025	20.7 (0.3)	32 (3)	11 (1)	168 (5)	64 (5)
HF-151	CT2-019 × CV2-018	21.3 (0.4)	38 (4)	13 (1)	169 (6)	56 (6)
HF-51	CT2-002 × CV2-018	19.7 (0.4)	28 (2)	10 (1)	184 (7)	74 (6)
HF-69	CT2-017 × CV2-046	20.7 (0.3)	28 (2)	10.4 (0.7)	149 (8)	63 (5)

*Numbers in brackets are the standard errors. W, Woondum Provenance.*

#### Klason Lignin Content

**FIGURE 1:**
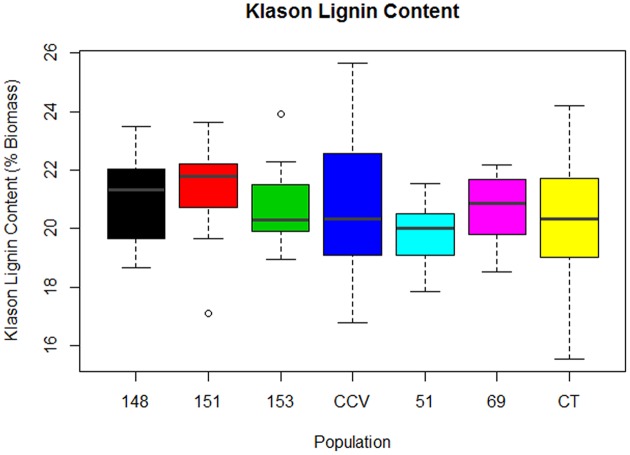
**Klason Lignin content of interspecies *Corymbia* hybrid populations and parental taxa at age 13 years, expressed as dry weight percentage of biomass.** Numeric codes for each population are provided in **Table 2**. Horizontal bars within each boxplot denote the population median with open circles representing outliers 1.5X outside the interquartile range. CCV, *Corymbia citriodora* subspecies *variegata*; CT, *Corymbia torelliana.*

Results from two-step acid hydrolysis show that *Corymbia* parental species (*Corymbia torelliana* and *C. citriodora* subspecies *variegata*) contained similar mean (M) values of Klason lignin as compared to their F1 interspecies hybrid counterparts. The parental species *Corymbia torelliana* (*M* = 20.4%, *SD* = 2.0%) and *C. citriodora* subspecies *variegata* (*M* = 20.9%, *SD* = 2.5%) contained greater variation within their Klason lignin content than each of the hybrid populations HF-148 (*M* = 21.0%, *SD* = 1.5%), HF-151 (*M* = 21.3%, *SD* = 1.5%), HF-153 (*M* = 20.7%, *SD* = 1.3%), HF-51 (*M* = 19.7%, *SD* = 1.2%), and HF-69 (*M* = 20.7%, *SD* = 1.2%) as shown in **Figure 1**.

Multiple linear regression of Klason lignin content as predicted by DBH and population was significant [*F*(13,92) = 5.02, *R*^2^ = 0.34, *P* = 7.2 × 10^-7^], with a significant interaction found between population and DBH [*F*(6,92) = 3.77, *P* = 0.002]. Within populations *C. citriodora* subspecies *variegata*, HF-148, HF-151, HF-153, and HF-51, Klason lignin content was predicted as equal to 19.0% +0.12 (DBH), where Klason lignin is expressed as percent total biomass and DBH is measured in cm. Within the *Corymbia torelliana* population, the slope and intercept was significantly different (*P* < 0.02) to other *Corymbia* populations, where *Corymbia torelliana* Klason lignin content was predicted as equal to 15.6% +0.32 (DBH). Additionally, within the hybrid population HF-69, the slope and intercept were significantly different (*P* < 0.02) than other *Corymbia* populations where Klason lignin content was predicted as equal to 21.0% -0.09 (DBH). Overall, in most populations Klason lignin content increased by 0.12% per cm increase of DBH. However, within the *Corymbia torelliana* population, Klason lignin content increased by 0.32% per cm increase of DBH, whereas Klason lignin content decreased by 0.09% per cm increase of DBH within hybrid population HF-69.

#### Glucan Content

**FIGURE 2:**
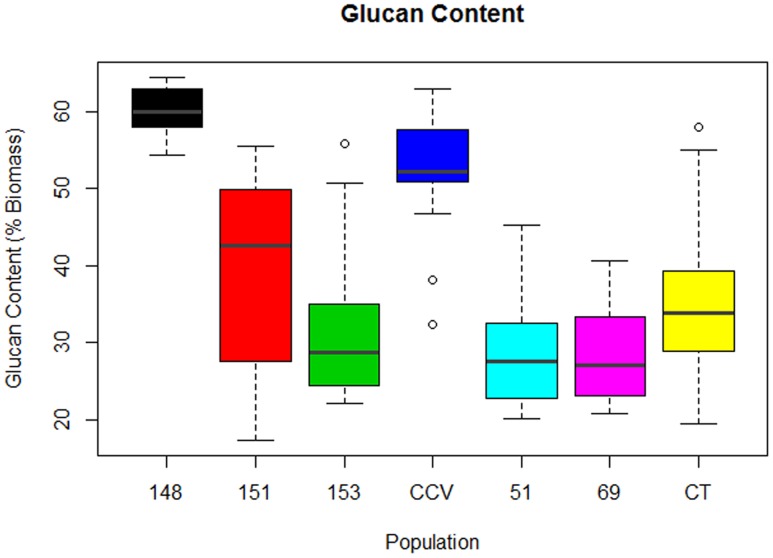
**Glucan content of interspecies *Corymbia* hybrid populations and parental taxa at age 13 years, expressed as dry weight percentage of biomass.** Numeric codes for each population are included within **Table 2**. Horizontal bars within each boxplot denote the population mean with open circles representing suspected outliers 1.5X outside the interquartile range. CCV, *Corymbia citriodora* subspecies *variegata*; CT, *Corymbia torelliana.*

Comparison of glucan content within each *Corymbia* population revealed significant differences among samples. Parental *Corymbia torelliana* (*M* = 35%, *SD* = 10%) and hybrid populations HF-151 (*M* = 38%, *SD* = 13%), HF-153 (*M* = 32%, *SD* = 11%), HF-51 (*M* = 28%, *SD* = 7%), and HF-69 (*M* = 28%, *SD* = 7%) contained similar mean and population variance for glucan content, while *C. citriodora* subspecies *variegata* (*M* = 52%, *SD* = 9%) and population HF-148 (*M* = 60.0%, *SD* = 3.4%) yielded much higher mean amounts of glucan, with population HF-148 containing the least variation (**Figure 2**). As such, these two populations were analyzed separately from other *Corymbia* population for the effect of DBH on glucan content.

Analysis of the *C. citriodora* subspecies *variegata* population and hybrid family HF-148 found no significant effect of DBH on glucan content, but a two-sided *t*-test found a significant difference [*t*(18.7) = 3.46, *P* = 0.002] in glucan content between *C. citriodora* subspecies *variegata* (*M* = 52%, *SD* = 8.2%) and hybrid family 148 (*M* = 60.0%, *SD* = 3.4%). MLR of the remaining *Corymbia* populations of glucan content as predicted by DBH and population was significant [*F*(5,63) = 4.36, *R*^2^ = 0.20, *P* = 0.002] with no significant interactions. The predicted glucan content was equal to 50% -0.7 (DBH), where glucan content is expressed as percent total biomass and DBH is measured in cm. Overall, glucan content decreased by 0.7% for every cm increase of DBH, and the glucan intercept (38%) for hybrid family HF-69 was significantly lower (*P* = 0.002) than other populations.

#### Xylan Content

**FIGURE 3:**
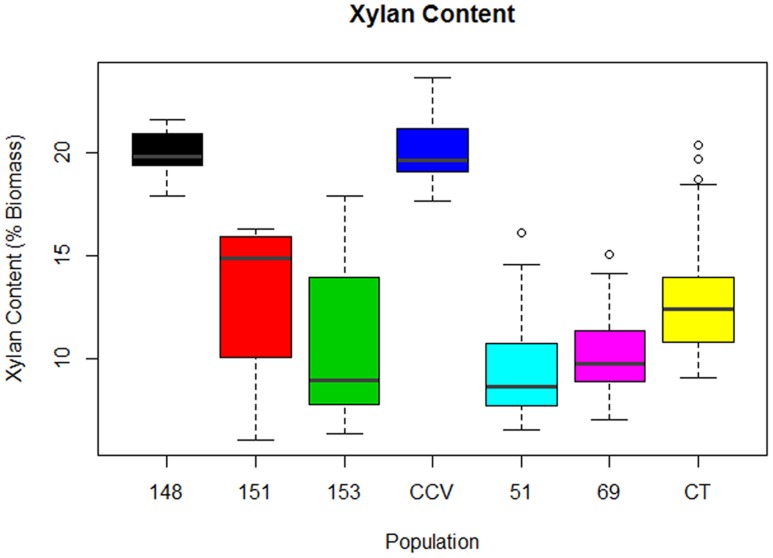
**Xylan content of interspecies *Corymbia* hybrid populations and parental taxa at age 13 years, expressed as dry weight percentage of biomass.** Numeric codes for each population are included within **Table 2**. Horizontal bars within each boxplot denote the population mean with open circles representing suspected outliers 1.5X outside the interquartile range. CCV, *Corymbia citriodora* subspecies *variegata*; CT, *Corymbia torelliana.*

Analysis of xylan content found the same trend as glucan content, with parental *C. citriodora* subspecies *variegata* (*M* = 20.2%, *SD* = 1.7%) and hybrid family HF-148 (*M* = 20.1, *SD* = 1.1%) containing highest mean xylan amounts. By comparison, the remaining *Corymbia* populations *Corymbia torelliana* (*M* = 13.4%, *SD* = 3.3%), HF-153 (*M* = 11%, *SD* = 4%), HF-151 (*M* = 13%, *SD* = 4%), HF-51 (*M* = 10%, *SD* = 3%), and HF-69 (*M* = 10.4%, *SD* = 2.7%) contained lower amounts of xylan (**Figure 3**). Due to the differences in xylan content, populations *C. citriodora* subspecies *variegata* and HF-148 were analyzed separately from other *Corymbia* population for the effect of DBH on xylan content. Analysis of *C. citriodora* subspecies *variegata* and 148 populations found no significant of DBH on xylan content and no significant differences between populations as tested with a two-tailed *t*-test (*P* > 0.05).

Multiple linear regression of the remaining *Corymbia* populations for xylan content as predicted by DBH and population was significant [*F*(5,59) = 5.41, *R*^2^ = 0.26, *P* = 0.0004] with no significant interactions. Xylan content was predicted as equal to 16% -0.3 (DBH), where xylan content is expressed as percent total biomass and DBH is measured in cm. In summary, xylan content decreased by 0.3% per cm increase of DBH. Although DBH significantly effected both Klason lignin content and structural polysaccharides, separate linear regressions of Klason lignin content as predicted by glucan and xylan content were not significant (*P* > 0.05), suggesting that polysaccharide content was not significantly affecting Klason lignin content within *Corymbia* populations.

### Enzymatic Saccharification

#### Total Glucose Production

**FIGURE 4:**
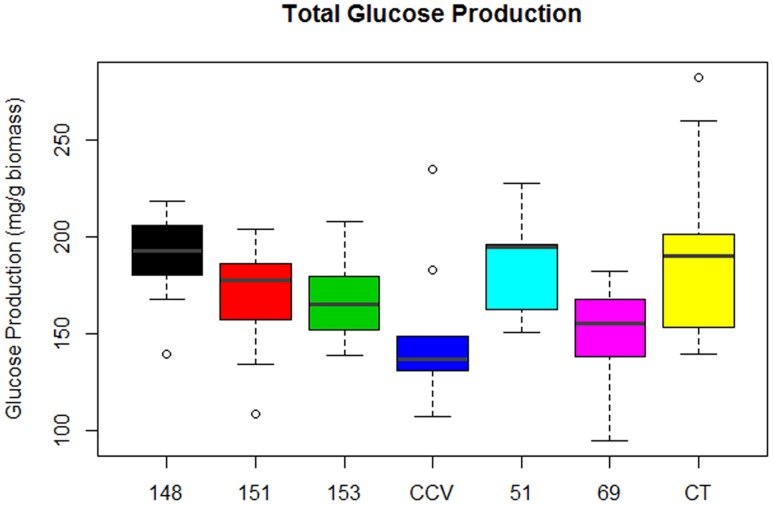
**Glucose released from enzymatic saccharification of *Corymbia* hybrids and parental taxa at age 13 years, expressed in mg of glucose per g of biomass.** Numeric codes for each population are included within **Table 2**. Horizontal bars within each boxplot denote the population mean with open circles representing suspected outliers 1.5X outside the interquartile range. CCV, *Corymbia citriodora* subspecies *variegata*; CT, *Corymbia torelliana.*

Analysis of total glucose production (mg glucose/g biomass) after enzymatic saccharification of hydrothermally pretreated biomass found significant differences in glucose release from populations of *Corymbia*. Comparison of population means found that parental *Corymbia torelliana* (*M* = 189, *SD* = 39) and hybrid populations HF-148 (*M* = 188, *SD* = 24) and HF-51 (*M* = 184, *SD* = 24) released the highest amounts of glucose from biomass, followed by hybrid populations HF-151 (*M* = 169, *SD* = 25), HF-153 (*M* = 168, *SD* = 21), *C. citriodora* subspecies *variegata* (*M* = 150, *SD* = 36) and HF-69 (*M* = 149, *SD* = 25) (**Figure 4**).

To investigate which biomass components significantly effected enzymatic saccharification of hydrothermally pre-treated samples, MLR of total glucose production was completed as predicted by population, Klason lignin content, and polysaccharide content (either glucan or xylan). Given the strong (Pearson) correlation between glucan and xylan (*r* = 0.93), each term was included separately into the saccharification MLR model to avoid multicollinearity.

**FIGURE 5:**
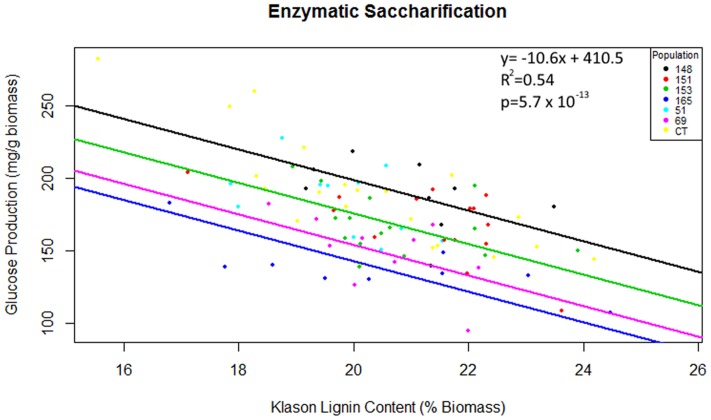
**Total glucose production as predicted by population and Klason lignin content.** Glucose production is expressed as mg of glucose released after 48 h of enzymatic hydrolysis per g of raw biomass and Klason lignin is expressed as a percentage of dry biomass. Numeric codes for each population are included within **Table 2**. Each colored regression line represents populations whose intercept is significantly different (*P* < 0.05) from the overall regression (black line). CCV, *Corymbia citriodora* subspecies *variegata*; CT, *Corymbia torelliana.*

Multiple linear regression of total glucose production as predicted by population, Klason lignin content and polysaccharide content (glucan or xylan) was significant [*F*(7,82) = 15.82, *R*^2^ = 0.54, *P* = 5.7 × 10^-13^], however, as glucan content, xylan content and interactions between explanatory variables were not significant (*P* > 0.05), these terms were removed from the final model. Total glucose production was predicted as equal to 410.5 -10.6 (Klason lignin), where glucose production was measured as mg of glucose released per g of pretreated biomass and Klason lignin was measured as percent total biomass. In summary, glucose production decreased by 10.6 mg for each percentage increase of Klason lignin content. The regression intercepts for populations HF-153 (387 mg/g), *C. citriodora* subspecies *variegata* (354 mg/g) and HF-69 (366 mg/g) were significantly lower (*P* < 0.02) than other *Corymbia* populations (**Figure 5**).

#### Glucan Conversion Efficiency

**FIGURE 6:**
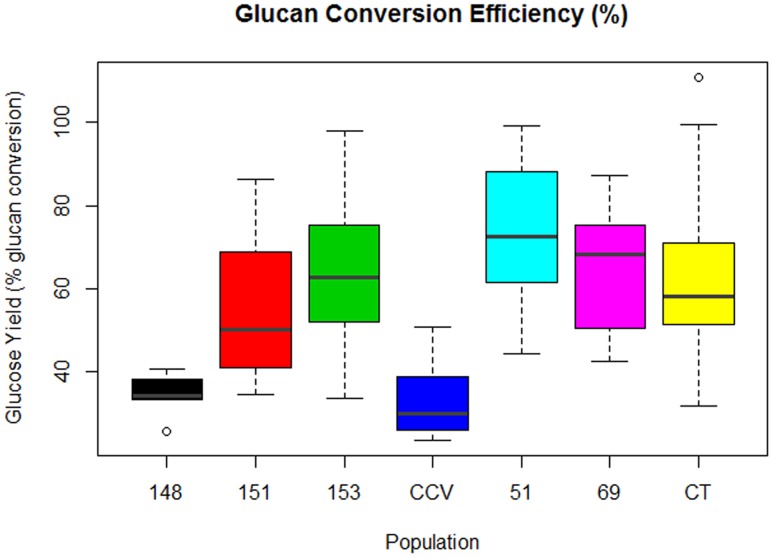
**Glucan conversion efficiency from enzymatic saccharification of *Corymbia* hybrids and parental taxa at age 13 years, expressed as a percentage of mg of glucose released per mg of glucan.** Numeric codes for each population are included within **Table 2**. Horizontal bars within each boxplot denote the population mean with open circles representing suspected outliers 1.5X outside the interquartile range. CCV, *Corymbia citriodora* subspecies *variegata*; CT, *Corymbia torelliana.*

Comparison of glucose conversion efficiency as expressed as a percentage of the theoretical conversion of anhydrous glucan (mg) to glucose (mg), showed significant differences among *Corymbia* populations (**Figure 6**). Population HF-51 (*M* = 74%, *SD* = 17%) underwent the most efficient conversion, followed by HF-153 (*M* = 64%, *SD* = 20%), HF-69 (*M* = 63%, *SD* = 16%), *Corymbia torelliana* (*M* = 62%, *SD* = 21%), HF-151 (*M* = 56%, *SD* = 19%), HF-148 (*M* = 35%, *SD* = 4%) and *C. citriodora* subspecies *variegata* (*M* = 33%, *SD* = 9%). MLR of glucose conversion as predicted by population and Klason lignin content was significant [*F*(6,76) = 7.97, *R*^2^ = 0.34, *P* = 1.2 × 10^-6^] with only population differences being significant. Within *Corymbia* populations, glucose conversion was significantly higher (*P* < 0.01) in populations *Corymbia torelliana*, HF-51, HF-151, HF-153, and HF-69 as compared to *C. citriodora* subspecies *variegata* and population HF-148.

### Additional Size Category Analysis

Although populations of *C. citriodora* subspecies *variegata* and hybrid family HF-151 did not contain trees with DBH beyond the large size class, the remaining *Corymbia* populations contained trees of much larger DBH. Given the significant effect of size on biomass composition, populations including larger trees (XL) and in the case of population HF-148, (XXL trees) (**Table 1**) were re-analyzed for Klason lignin, glucan and xylan content to see whether the same trends continued at larger DBH sizes.

#### Klason Lignin Content

Multiple linear regression of Klason lignin content as predicted by population and DBH was significant [*F*(9,93) = 9.22, *R*^2^ = 0.42, *P* = 7.1 × 10^-10^] with a significant interaction between population and DBH [*F*(4,93) = 2.50, *P* = 0.04]. With the inclusion of the larger trees, Klason lignin content was predicted as equal to 19.2% +0.11 (DBH), where Klason lignin content was expressed as percent total biomass and DBH was measured in cm. Overall, Klason lignin content increased by 0.11% for every cm increase of DBH, whereas hybrid family HF-69 Klason lignin increased by 0.02% for each cm increase in DBH (*P* = 0.02).

#### Glucan Content

Multiple linear regression of the *Corymbia* populations for glucan content (again excluding population HF-148) as predicted by population and DBH was also significant [*F*(7,65) = 3.44, *R*^2^ = 0.19, *P* = 0.003], with a significant interaction found between population and DBH [*F*(3,65) = 3.92, *P* = 0.01]. With the inclusion of the larger trees, glucan content was predicted as equal to 48% -0.9 (DBH), where glucan content is expressed as percent biomass and DBH is measured in cm. Overall, glucan content decreased by 0.9% for each cm increase of DBH, whereas hybrid family HF-69 glucan content increased by 0.3% per cm increase of DBH (*P* = 0.001).

#### Xylan Content

Multiple linear regression of xylan content within *Corymbia* populations (with the exclusion of HF-148) as predicted by population and DBH was significant [*F*(7,58) = 6.08, *R*^2^ = 0.35, *P* = 2.4 × 10^-5^], with a significant interaction between population and DBH [*F*(3,61) = 3.07, *P* = 0.03]. With the inclusion of the larger tree populations, xylan content was predicted as equal to 17% -0.4 (DBH), where xylan content is expressed as percent biomass and DBH is measured in cm. In summary, xylan content decreased by 0.4% for each cm increase of DBH. Within hybrid family 69, xylan content was predicted as equal to 11% with less effect as DBH increases (-0.1% per cm increase, *P* = 0.01).

## Discussion

In this study, determination of biomass composition of *Corymbia* F1 interspecies hybrids and parental species was completed using the NREL Laboratory Analytical Procedure-Determination of Structural Carbohydrates and Lignin in Biomass (Sluiter et al., 2011), which corrects for ash content within acid insoluble residue measured gravimetrically after pyrolysis at 575°C. During testing and optimization of this procedure, ash content within *Corymbia* samples was below an amount that could be reliably measured with an analytical balance, resulting in a protocol modification that shortened the pyrolysis step to clean crucibles before the next use. Ash content is detrimental to liquid fuel conversion processes (as non-biodegradable residue) as well as negatively affecting the calorific value of wood and plant processing costs from thermo-chemical conversion (McKendry, 2002; Jørgensen et al., 2007). *Eucalyptus* and *Corymbia* have been characterized as possessing low ash content (<1%), (Guerrero et al., 2005; Magalhães et al., 2011; Çetrinköl et al., 2012), which decreases with tree age (Kumar et al., 2010).

If an advanced biofuel feedstock to be considered sustainable, it must possess a growth rate that warrants continued economic harvesting of that crop (Hinchee et al., 2009). Despite the advantages of lignocellulose for biofuel production, the presence of lignin and the biomass’ natural recalcitrance are substantial barriers to overcome, before this can be realized. During the growth and expansion of the plant cell wall, shifting carbon resources can simultaneously increase lignin content while decreasing polysaccharide content. This has been demonstrated through transgenic manipulation of lignocellulose biosynthesis. While in some instances, lignin reduction results in increased cellulose content, biomass and growth rate (Hu et al., 1999), disruption of lignin biosynthesis typically results in negative pleiotropic effects on growth and form. For example, suppression of the LIM domain transcription factor in *Eucalyptus camaldulensis*, an positive regulator of several lignin biosynthesis genes, resulted in a transgenic lines with reduced lignin content (17% as compared to 24%-wild-type [WT]) that frequently dropped upper leaves (Kawaoka et al., 2006). Specific targeting of the phenylpropanoid cinnamoyl-CoA reductase (*CCR)* gene in transgenic poplars, produced trees with less lignin (17% vs. 21%-WT) that that had significantly reduced growth (height, DBH, and growth rate) (Leplé et al., 2007). Additionally, disruption of lignin biosynthetic pathways often accompanies increased deposition of phenolics and extractives within wood tissue resulting in discolouration. Down-regulation of 4-coumarate:coenzymeA ligase (*4CL*) in poplar also results in reduced total lignin content and discolouration of xylem tissues, with biomass and leaf area reduced by half as compared to WT (Voelker et al., 2011). Adequate lignification of xylem vessels allows long distance transport of water through maintenance of internal water tension. Irregular xylem formation causes vasculature collapse, inadequate water transport, and weakened carbon sink strength (Coleman et al., 2008). Alternatively, overexpression of gibberellin 20-oxidase (a precursor to the gibberellin hormone) in hybrid poplars resulted in trees with faster growth in height and DBH, increased biomass, and more numerous and longer xylem fibers (Eriksson et al., 2000). Considering the increased biomass production of the *Corymbia* hybrid populations (Lee et al., 2009), it is reasonable to expect increased lignification of xylem tissue correlates with growth, consistent with wood samples taken across each size category (**Table 1**).

Enzymatic saccharification of *Corymbia* biomass demonstrates the strong negative effect lignin content on enzymatic hydrolysis. This is attributed to the structure of lignin physically inhibiting enzymatic access to cellulose microfibrils, forming cross-linkages with hemicellulose, and lignin non-specifically binding and immobilizing cellulases (Yu et al., 2011; Zhao et al., 2012; Leu and Zhu, 2013). Without delignification, up to 70% of cellulases remain immobilized within lignin (Berlin et al., 2005; Jørgensen et al., 2007). This has resulted in development of a variety of pretreatments for eucalypt biomass, to increase efficiency of enzymatic hydrolysis (Yu et al., 2010; Silva et al., 2011; Papa et al., 2012; Santos et al., 2012; Yáñez-S et al., 2013; Zhang C. et al., 2015). Lignin removal also creates pores in the cell wall through which cellulases can gain access to cellulose microfibrils (Yu et al., 2011). The pretreatment method for this dataset was hydrothermal (pressurized hot-water), which does not remove lignin but solubilizes hemicellulose, and disrupts the cellulose-hemicellulose-lignin complex. In this study, nor glucan or xylan content significantly impacted total glucose production from biomass. Cellulose, the primary donor of glucose during saccharification, resists enzymatic hydrolysis through hydrogen bonding and microfibril crystallinity. The highly compact cellulose polymer is hydrophobic, so only the hydrophilic ends of the microfibril are susceptible to enzymatic attack. Without disruption of the microfibril structure (normally achieved through energy intensive ball-milling) which increases porosity and promotes cellulose accessibility, glucan content independently considered may not significantly affect saccharification (Leu and Zhu, 2013) or ethanol production during simultaneous saccharification and fermentation (Vinzant et al., 1997). Additionally, an increase in hemicellulose content has been demonstrated to disrupt cellulose crystallinity in *Miscanthus*, thereby increasing enzymatic hydrolysis after acidic and alkaline pretreatment (Xu et al., 2012), the opposite effect has been demonstrated in Poplar transgenic experiments, where disruption of glycosyltransferase GAUT12 resulted in transgenic trees with less xylan (17–30% reduction) and increased saccharification yield (4–8% increase in glucose recovery) without a significant reduction in lignin content (Biswal et al., 2015). Future studies of this nature would benefit from the investigation of the transcriptome within the natural trait extremes within populations that cannot yet be transformed in order to discern the genetic mechanisms by which trees compensate for an increased growth rate.

Within *Corymbia* samples, the highest conversion efficiency was achieved within population HF-51 (*M* = 74%), with populations HF-153, HF-51, and *Corymbia torelliana* all containing samples that approached 100% conversion (**Figure 6**). While higher glucan conversion has been achieved in the literature (Sykes et al., 2015), our intent was to maximize the relative differences among parental species and F1 hybrid populations. *C. citriodora* subspecies *variegata* and HF-148 underwent the least efficient conversion of glucan to glucose, unsurprising considering the fixed enzyme dosage, however, HF-148 released the highest mean glucose amount from biomass, a promising result for future investigation with alternative pretreatments designed to increase cellulose accessibility and saccharification.

Given the economic importance of eucalypt taxa for industrial processes such as pulp and paper, their biomass composition has been well researched (**Table 3**). Klason lignin content among the *Corymbia* populations is consistent with values found in the literature and while acid soluble lignin was not measured here, it is reasonable to expect a similar range (2–4%) that would also likely have a detrimental effect on saccharification and subsequent fermentation (Ximenes et al., 2010). While the xylan content among *Corymbia* populations is consistent with those found in the literature for eucalypts, mean glucan content within *Corymbia torelliana* and hybrid families HF-153, HF-51, and HF-69 are low in comparison to HF-151, *C. citriodora* subspecies *variegata* and HF-148.

**Table 3:** Composition of major structural components of eucalypt biomass.

	Composition (%)				
Species	Glucan	Xylan	Klason Lignin	Acid Soluble Lignin	Reference
*E. globulus*	46.1	14	20.9	3.0	Santos et al., 2011
*E. nitens*	41.8	15.9	22.3	3.2	
*E. urophylla* × *E. grandis*	48.5	10.7	24.5	2.1	
*E. grandis*	44.9	11.4	26.2	–	Yu et al., 2010
*E. globulus*	46.3	16.6	23	3.5	Garrote et al., 2007
*E. grandis*	44.6	15.33	25.8	–	Emmel et al., 2003
*E. dunnii*	47.5	17.31	27	3.4	McIntosh et al., 2012
*C. citriodora* subspecies *variegata*	48.5	17.1	24.36	4.19	
*E. urophylla* × *E. grandis*	59	–	20	–	Shinya et al., 2016

While mean glucan content in *C. citriodora* subspecies *variegata* and HF-148 samples is higher than other *Corymbia* populations or other literature values, the result is consistent with α-cellulose content (cellulose which remains insoluble) within elite *Eucalyptus* hybrids (*E. urophylla* × *E. grandis*) (Shinya et al., 2016). In their study Shinya et al. (2016) evaluated the wood properties of 918 hybrids and selected two genotypes (AM380 and AM063) for their extreme Klason lignin content (35 and 20%, respectively). Further characterization of AM063 biomass estimated its α-cellulose content was 59% dry weight (native wood). Similarly, the α-cellulose content of genotype AM380 was estimated at 48%, suggesting a negative correlation between lignin and cellulose. While the results glucan content from *C. citriodora* subspecies *variegata* and HF-148 were consistent with Shinya et al. (2016), the negative (Pearson) correlation between Klason lignin and glucan content within this dataset (*r* = -0.1) was not significant (*P* > 0.05). Despite cellulose being the principle contributor of glucan released from lignocellulose, the primary cell wall of woody species also contains xyloglucan, a matrix polysaccharide which contains glucan and xylan residues (Carpita and Gibeaut, 1993; Harris and DeBolt, 2010). If the high % dry weight of xylan within *C. citriodora* subspecies *variegata* and HF-148 can be attributed to xyloglucan, then it would be reasonable to expect that glucan content could also be increased.

**FIGURE 7:**
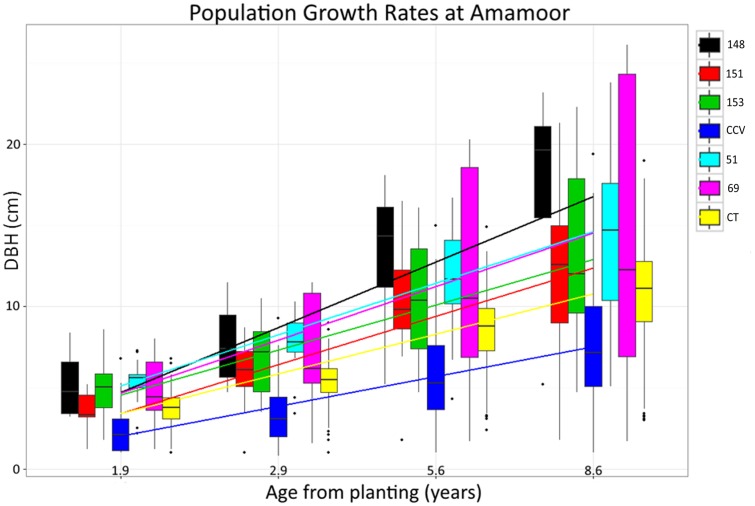
**Historical *Corymbia* growth measurements taken from the Amamoor plantation.** Measurements of diameter breast height (DBH) were taken at years 1.9, 2.9, 5.6, and 8.6 from planting.

F1 generation hybrids are typically intermediate with regard to parental trait values, but occasionally hybrids will either resemble one parent or exceed both (Rosenthal et al., 2002; Barbour et al., 2003). Despite lignin content of hybrid populations being intermediate to parental species, structural polysaccharide content in *Corymbia* hybrids HF-153, HF-51, HF-69 resembled the *Corymbia torelliana* parental species, whereas HF-151 was typically intermediate, and HF-148 was transgressive for glucan content (**Figure 2**). Other investigation of stem and leaf attributes among controlled-cross F1 *Corymbia torelliana* × section *Maculatae* crosses (to which *C. citriodora* subspecies *variegata* belongs), hybrids typically resembled the *Corymbia torelliana* maternal parent or were transgressive (Abasolo et al., 2012). Based on maternal parentage and the moderate to high heritability of cellulose content (*h*^2^ = 0.42–0.86) (Kube et al., 2001; Apiolaza et al., 2005; Stackpole et al., 2010) it would be expected that *Corymbia* hybrids with shared genetics (HF-148 and HF-153) should be similar in structural polysaccharide content. This, however, is not the case. Additionally, for the hybrid families that have at least one parent in common (HF-148 and HF-69; HF-51 and HF-151) parentage of the common male pollen parent among the hybrids failed to elucidate genetic patterns underlying polysaccharide content. Population HF-148 is clearly distinguished from the other hybrids by having transgressive glucan content, while historical investigation of data collected from the Amamoor plantation site also confirms heterosis for growth rate (**Figure 7**). Between ages 1.9 and 8.6, each hybrid family at Amamoor, in terms of DBH, outperformed parental species with HF148 distinguished having the fastest growth beyond age 2.9.

Specific consideration of hybrid family HF-69 found each of its traits relating to biomass composition responded significantly different from other *Corymbia* populations. With the inclusion of the XL DBH tree data, Klason lignin, glucan, and xylan content as predicted by DBH of HF69 was less impacted by increasing tree size. Interestingly, evaluation of historical growth records from the Amamoor plantation also indicate HF-69 displayed an odd growth habit, displaying nearly double the variation in DBH of any other *Corymbia* population at age 8.6 (**Figure 7**). While HF-69 was not a top performer regarding total glucose production (*M* = 149 mg/g biomass) or glucan to glucose conversion (*M* = 63%), its biomass stability and large growth variation for selection and improved breeding may be desirable for other forest industries, such as timber production, where product consistency is highly regarded.

## Conclusion

As advanced future biofuel feedstocks require a high rate of growth to justify harvesting at an industrial scale and efficient deconstruction and conversion is dependent on biomass composition, a key consideration for a renewable feedstock is the impact of growth on lignocellulose formation. This investigation identified that in response to growth in *Corymbia* populations, major structural components of biomass were significantly impacted by tree size, shifting toward increased recalcitrance through increased Klason lignin content and deceased polysaccharide content. This research suggests that fast growing trees harvested under fast rotations would be best suited for lignocellulosic biofuel production. Given current forestry management practices involve thinning trees planted at a high stocking rate to promote growth in high value trees, traditional forestry and bioenergy applications could be combined if thinned trees are removed for biofuel use before lignification is complete.

## Author Contributions

AH: Responsible for overall experimental design, sample collection, analysis and manuscript writing. DL: Responsible for overall experimental design, sample collection, interpretation of results and manuscript editing. JL: Responsible for overall experimental design, processing samples for analysis, interpretation of results and manuscript writing. GP: Responsible for two-step acid hydrolysis design, collecting and interpreting composition data, and manuscript writing. JG: Responsible for high-throughput saccharification design, collecting and interpreting glucose data, and manuscript editing. LC: Responsible for high-throughput design and collection of structural polysaccharide data and manuscript editing. FA: Responsible for hydrolysis and saccharification design, interpretation of results and manuscript editing. SS: Responsible for two-step acid hydrolysis design, interpretation of results and manuscript editing. BS: Responsible for hydrolysis and saccharification design, interpretation of results and manuscript editing. RH: Responsible for overall experimental design, interpretation of results and manuscript writing. All authors have agreed on the final version of this manuscript and are accountable for the research therein.

## Conflict of Interest Statement

The authors declare that the research was conducted in the absence of any commercial or financial relationships that could be construed as a potential conflict of interest.

## Footnotes

1 www.advancedmaterials.us

2 www.gelifesciences.com
